# Downregulation of ANP32B exerts anti-apoptotic effects in hepatocellular carcinoma

**DOI:** 10.1371/journal.pone.0177343

**Published:** 2017-05-09

**Authors:** Yoshinori Ohno, Mitsuhito Koizumi, Hironao Nakayama, Takao Watanabe, Masashi Hirooka, Yoshio Tokumoto, Taira Kuroda, Masanori Abe, Shinji Fukuda, Shigeki Higashiyama, Teru Kumagi, Yoichi Hiasa

**Affiliations:** 1 Department of Gastroenterology and Metabology, Ehime University Graduate School of Medicine, Toon, Ehime, Japan; 2 Department of Biochemistry and Molecular Genetics, Ehime University Graduate School of Medicine, Toon, Ehime, Japan; 3 Division of Cell Growth and Tumor Regulation, Proteo-Science Center (PROS), Ehime University Graduate School of Medicine, Toon, Ehime, Japan; Institute of Biochemistry and Biotechnology, TAIWAN

## Abstract

The acidic (leucine-rich) nuclear phosphoprotein 32 family member B (ANP32B), a highly conserved member of the acidic nuclear phosphoprotein 32 (ANP32) family, is critical for the development of normal tissue. However, its role in the development of hepatocellular carcinoma (HCC) is controversial. In this study, we elucidated the role of ANP32B in HCC cell lines and tissues. ANP32B expression in HCC cell lines was modulated using siRNA and ANP32B expression plasmids and lentiviruses. The levels of apoptosis-related proteins were analyzed by real-time RT-PCR and Western blotting. The expression of ANP32B in tissues from patients with HCC was investigated using real-time RT-PCR and immunohistochemistry. ANP32B knockdown by siRNA altered the expression of apoptosis-related proteins in HCC cell lines and reduced the expression of cleaved forms of caspase 3 and caspase 9, but not that of caspase 8, in HCC cells cultured with the pro-apoptotic agent staurosporine. Phosphorylated Bad was upregulated, whereas Bak was downregulated. Moreover, ABT-737, which binds to and inhibits anti-apoptotic proteins of the Bcl-2 family, rendered HCC cells resistant to apoptosis induced by ANP32B silencing. Conversely, ANP32B overexpression decreased Bad phosphorylation and upregulated Bak, but did not induce apoptosis because Bax expression was downregulated. In tissues from patients with HCC, a low tumor/non-tumor ratio of ANP32B mRNA expression was related to advanced UICC stage (p = 0.032). TUNEL-positive cells were observed in parallel with ANP32B expression in HCC tissues. ANP32B modulates Bad phosphorylation as well as Bak and Bax expression, resulting in regulation of apoptosis in HCC. These findings indicate the potential value of ANP32B as a therapeutic target for HCC.

## Introduction

Hepatocellular carcinoma (HCC) is one of the most common malignancies worldwide [1]. The prognosis of HCC has been significantly improved in recent years by earlier diagnosis and more effective treatments [2]. However, patients diagnosed at an advanced stage or with progression after locoregional therapy present a poor prognosis [3]. Patients with HCC present with two major characteristics. One is that HCC frequently leads to multi-center carcinogenesis, and relapse often occurs after locoregional therapy. The second is that HCC frequently accompanies liver cirrhosis. There is a risk of liver failure with repetitive or invasive treatment. Markers to predict the prognosis and treatment efficacy are important in selecting the treatment method.

In HCC, acidic leucine-rich nuclear phosphoprotein 32 family member A (ANP32A) has been reported to promote tumor growth and to be a marker of poor prognosis [4,5]. Moreover, proteomic analysis suggested that ANP32A is a potential marker in HCC tissues [6]. In the liver, ANP32A acts as a growth factor [7] and protects hepatocytes from injury induced by CCL4 [8]. ANP32A also attenuates oxidative injury and fibrosis induced by ethanol feeding [9].

ANP32 is a novel nuclear protein and a member of the highly conserved acidic leucine-rich nuclear phosphoprotein 32 (ANP32) family, whose members, including ANP32A, B, C, D, E, F, G, and H, are characterized by a C-terminal acidic tail and a N-terminal leucine-rich repeat (LRR) [10–13]. ANP32 members have physiologically diverse functions, including chromatin modification and remodeling, apoptotic caspase modulation, protein phosphatase inhibition, and regulation of intracellular transport [10]. In particular, ANP32A, B, and E are the most important and act cooperatively or in an opposite manner with each other [10]. However, few studies have investigated the role of ANP32B or ANP32E in HCC. Recently, Reilly et al. [14] reported that ANP32B is the most critical for normal development by comparing the effects of ANP32B deficiency to those of ANP32A or ANP32E deficiency in mice. However, the role of ANP32B in the growth and prognosis of human HCC remains unknown.

We confirmed that ANP32B is expressed in HCC tissues using real-time RT-PCR, Western blotting, and immunohistochemistry. Focusing on ANP32B, we considered that ANP32B may play a critical role in HCC, similar or opposite to that of ANP32A. The present study aimed to clarify the role of ANP32B in HCC cell lines and tissues from patients with HCC.

## Materials and methods

### Cell culture and transfection

Three liver cancer cell lines, Huh7, HLE, and HepG2 (Japanese Collection of Research Bioresources, Osaka, Japan), were grown and maintained in Dulbecco’s modified Eagle medium (DMEM; Life Technologies) supplemented with 10% fetal bovine serum (Life Technologies) and 1% penicillin. Cells were maintained at 37°C in a humidified atmosphere of 5% CO2 and 95% air, and the culture medium was changed three times per week.

### RNA interference

Small interfering RNA (siRNA) targeting regions of the human ANP32B gene (siANP32B: SASI_Hs02_00341480, si2ANP32B: SASI_Hs02_00341479) (Sigma, Tokyo, Japan) and control siRNA (GE Healthcare, Tokyo, Japan) were designed and produced. Huh7, HLE, and HepG2 cells at 70% confluence in 6-well plates were transfected with 50 pM siRNA using Lipofectamine RNAiMax (Life Technologies). The knockdown efficiency was validated by quantitative PCR and western blot analyses.

### RNA extraction, cDNA synthesis, and real-time RT-PCR

Total RNA was extracted with TRIzol reagent (Life Technologies). The RNA was reverse transcribed using RT-PCR kits (Applied Biosystems, Foster City, CA, USA) with an oligo d(T)16 primer under standard conditions. Real-time PCR was performed using a 7500 real-time PCR system (Applied Biosystems) and 2 μL of purified cDNA product, 1 μL of primer, and 10 μL of Universal Master mix II (Applied Biosystems). Commercial ANP32B (Hs.730654), BAD (Hs.370254), BAK (Hs, 485139), and glyceraldehyde phosphate dehydrogenase (GAPDH) (Hs.544577) (Applied Biosystems) primer sets were used for PCR amplification under the conditions recommended by the manufacturer. GAPDH served as an internal reference gene. The relative mRNA expression levels of host genes divided by the amount of GAPDH mRNA were evaluated by statistical analysis.

### PCR array analysis and antibody array

To better understand the role of ANP32B, the RT2 Profiler PCR array system (QIAGEN, Tokyo, Japan) and the LightCycler system (Roche) were used according to the manufacturers’ instructions. Threshold cycle values were analyzed using web-based PCR array data analysis software (http://pcrdataanalysis.sabiosciences.com/pcr/arrayanalysis.php). Of the kits supplied with the PCR array system, the Apoptosis PCR array (QIAGEN, Tokyo, Japan) was used. The Pathscan (Cell Signaling, Danvers, MA, USA) intracellular signaling array kit (chemiluminescent Readout) is an antibody array based upon the sandwich immunoassay principle.

### Western blotting

For Western blotting, 20 μg of protein was applied to the lanes of 4% to 12% Bis-Tris gels (Life Technologies) for electrophoresis, blotted onto Immobilon-P membranes (Millipore, Bedford, MA, USA) after electrophoresis, and incubated with the relevant primary antibody: anti-beta-actin (Chemicon, Temecula, Ca, USA); anti-PHAPI1/APRIL (ANP32B) (Abcam); anti-GFP (MBL, Nagoya, Japan); anti-Bad (9292) anti-phospho-Bad (5284), anti-Bcl-2 (2870), anti-Bak (12105), anti-Bcl-xL (2764), anti-Bcl-2 (2870), anti-phospho-Akt (4060), anti-Akt (9272), anti-caspase-3 (9662), anti-caspase-8 (9746), and anti-caspase-9 (9502) antibodies (Cell Signaling). Appropriate species-specific conjugated secondary antibody kits were commercially obtained (GE Healthcare). Proteins were detected using the ECL prime Kit (GE Healthcare) with an ImageQuant LAS4000 system (GE Healthcare).

### Apoptosis assays

Two cell lines (huh7 and HLE) were grown overnight and transfected with ANP32B siRNA or control siRNA. The cells were treated with staurosporine (0.8 μM for Huh7 cells and 0.04 μM for HLE cells) (Wako, Tokyo, Japan) dissolved in DMSO (Sigma) or an equivalent volume of DMSO alone, and cultured for 12 h. The cells were alternatively treated with etoposide (600 μM for Huh7 cells) (Wako, Tokyo, Japan) dissolved in DMSO or an equivalent volume of DMSO alone, and cultured for 24 h. Following treatment, the attached cells were collected, and apoptosis was assayed using the Annexin V-PE or APC (BD Biosciences, San Jose, CA, USA) and 7-amino-actinomycin D (7-AAD) (TONBO biosciences, San Diego, CA, USA) assays according to the manufacturer’s protocol. Stained cells were examined using a FACSCalibur system (Becton Dickinson, Franklin Lakes, NJ, USA) and analyzed with FlowJo software (TreeStar Corporation, Ashland, OR, USA). The cells were treated with ABT-737 (Funakoshi, Tokyo, Japan) and cultured for 4–7 h (4 h for siRNA or 7 h for overexpression), or treated with the pan-caspase inhibitor Z-VAD-FMK (Selleck Chemicals, Houston, TX, USA).

To detect cells with oligonucleosomal DNA breaks, the HCC specimens were also subjected to terminal deoxynucleotidyl transferase-mediated 2′-deoxyuridine 5′ -triphosphate nick-end labeling (TUNEL) staining using a Mebstain apoptosis TUNEL kit II (MBL, Nagoya, Japan).

### ANP32B overexpression by plasmids and lentiviruses

ANP32B overexpression was performed using a plasmid that expressed the ANP32B gene. Huh7 cells were transfected with pEZ-M03-ANP32B-GFP (GeneCopoeia, Rockville, MD, USA). The GFP plasmid (pEZ-M03-GFP) was used as the control plasmid. Each plasmid (4 μg/mL) was transfected into Huh7 and HLE cells at 70% confluence using Lipofectamine LTX (Life Technologies).

ANP32B overexpression was additionally performed by using a lentiviral system [15]. Lentiviruses used in this study were constructed by inserting cDNA encoding ANP32B-GFP or GFP or ANP32B into a lentiviral expression vector, CSII-CMV-MCS-IRES2-Bsd. The lentiviral vectors were then transfected into HEK 293T cells together with two packaging plasmids, pCAG-HIVgp and pCMV-VSV-G-RSV-Rev. Huh7 cells were infected with the ANP32B-overexpressing lentiviruses and the infected cells were maintained.

### Patients and liver specimens

Liver specimens of HCC and corresponding non-tumor tissues (NT) were obtained from 31 patients who underwent surgery at Ehime University Hospital. Samples of freshly resected liver specimens were incubated with RNAlater (Life Technologies, Carlsbad, CA, USA) overnight at 4°C and then frozen and stored at -80°C until used. Additional samples were placed on dry ice, frozen immediately, and then stored at -80°C until used for protein extraction. We performed real-time quantitative RT-PCR for ANP32B mRNA from frozen paired samples derived from patients with HCC. Pathological stages were divided into stages I + II and III + IV. Written informed consent was obtained from all patients. The study protocol conformed to the ethical guidelines of the Declaration of Helsinki and was approved by the Institutional Review Board at Ehime University Hospital (Approval No. 1412015). This study was registered in the University Hospital Medical Information Network (UMIN) Clinical Trials Registry (registration number 000016944).

### Immunohistochemistry of HCC specimens

Liver specimens of HCC and non-tumor tissues were fixed in formalin. Sections (3-μm-thick) were cut from each block, and adjacent sections were stained using standard hematoxylin and eosin (H&E) and immunohistochemical staining techniques. Paraffin-embedded samples were dewaxed and rehydrated. The primary anti-PHAPI1/APRIL (ANP32B) monoclonal antibody (Abcam, Tokyo, Japan) was diluted 1:50 and incubated with the sections at 4°C overnight. Tissue sections were treated with peroxidase-labeled secondary antibody (histofine Simplestain Max PO; Nichirei, Tokyo, Japan) for 1 h at room temperature and incubated with Simple Stain DAB Solution (Nichirei).

### Pathological analyses

Slides were imaged in bright field using the NanoZoomer Digital Pathology System (Hamamatsu Photonics, K.K., Japan). Expression of ANP32B protein and TUNEL staining in human HCC specimens were evaluated as the average count of ANP32B-positive cells in 10 image fields (×400) and the average count of TUNEL-positive cells in 10 image fields (×200).

### Statistical analysis

All statistical analyses were performed using JMP 11.0 (SAS Institute, Tokyo, Japan). Data expressed are the mean and standard error (SE) or the mean and standard deviation (SD). Continuous variables were analyzed using the Mann-Whitney U test. Categorical variables were analyzed using the chi-squared test. Correlations between two variables were evaluated by using Pearson product-moment correlation coefficients. p < 0.05 was considered to indicate statistical significance.

## Results

### ANP32B knockdown suppressed apoptosis in HCC cell lines

**Fig 1:**
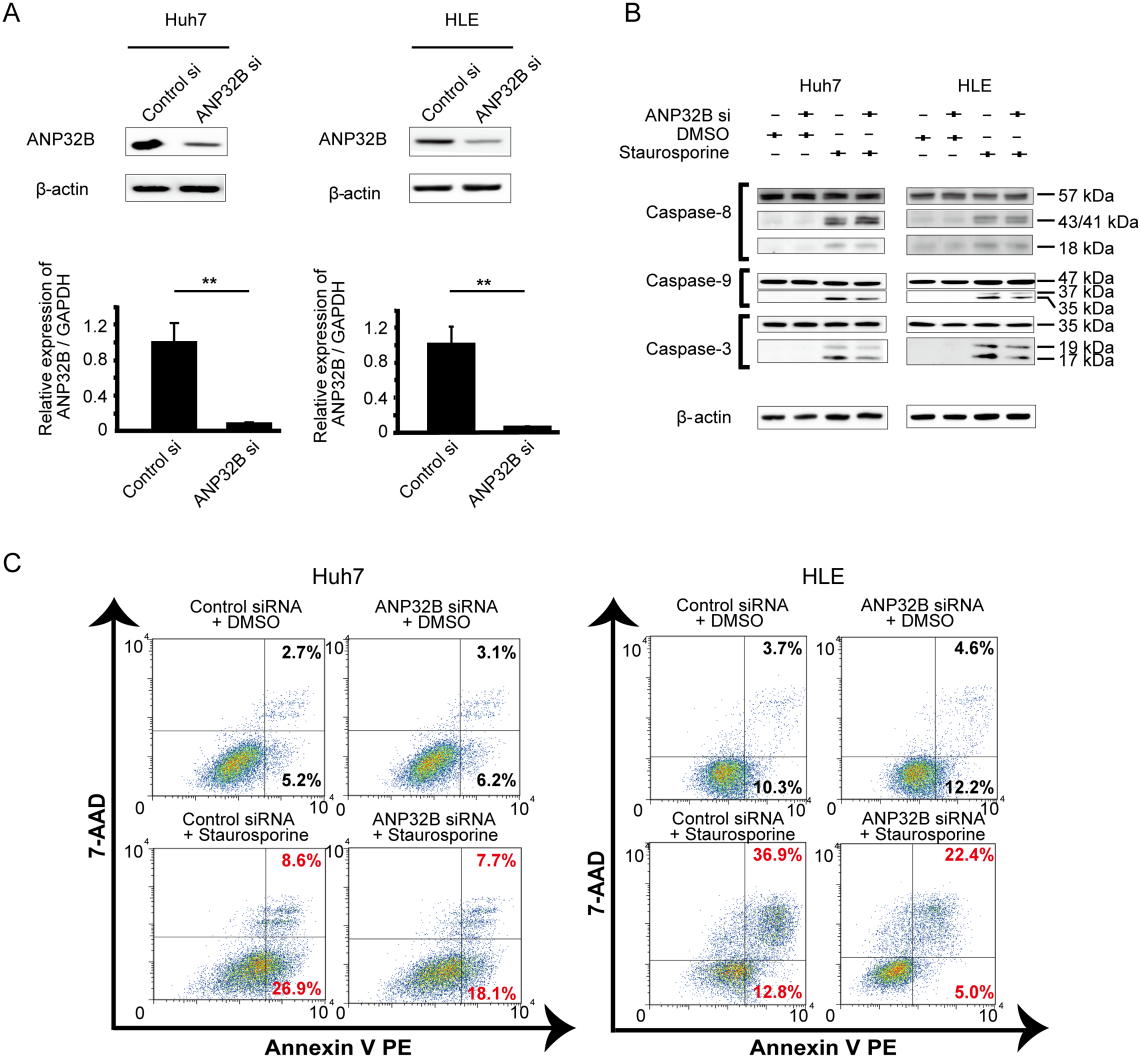
ANP32B knockdown by siRNA suppressed apoptosis in HCC cell lines. **(A)** Cells were transfected with either ANP32B or control siRNA in Huh7 and HLE cells. The knockdown efficiency was validated by Western blotting and real-time RT-PCR. β-actin was used as an internal control. ANP32B mRNA levels were quantified by real-time RT-PCR. Mean ± SEM of six replicates. **p < 0.01. **(B)** ANP32B was knocked down by siRNA, and cells were cultured with the pro-apoptotic agent staurosporine, for 12 h. The expression of cleaved forms of caspase 3, caspase 8, and caspase 9 proteins was analyzed by Western blotting. **(C)** The apoptotic effect of ANP32B knockdown by siRNA in Huh7 and HLE cells cultured with or without staurosporine was analyzed using flow cytometry. Control siRNA-transfected cells cultured with staurosporine were used as the positive control. Apoptotic cells were determined as positively labeled with annexin V, and necrotic cells were determined as positively labeled with 7-AAD.

The human HCC cell lines Huh7 and HLE were used for in vitro studies. Jurkat cells, which express ANP32B, were used as a positive control. We performed Western blotting to evaluate ANP32B protein expression and confirmed that ANP32B was expressed in all HCC cell lines (S1A Fig). To investigate the role of ANP32B, we knocked down ANP32B expression in Huh7 and HLE cell lines by siRNA. ANP32B was most efficiently knocked down by ANP32B siRNA until day 3 after transfection in Huh7 cells (S1B Fig). Real-time RT-PCR and Western blotting revealed that ANP32B siRNA efficiently knocked down ANP32B expression in Huh7 and HLE cells (Fig 1A).

ANP32B has been reported to relate to apoptosis in leukemic cells. However, whether it acts as an anti-apoptotic or a pro-apoptotic factor is controversial [16–18]. Thus, we focused on the apoptotic pathway in liver cancer. We cultured Huh7 and HLE cells with staurosporine, a pro-apoptotic agent. Knocking down ANP32B resulted in reduced expression of cleaved forms of caspase 3 and caspase 9, but not of caspase 8, in HCC cells cultured with staurosporine (Fig 1B). We next analyzed apoptosis using annexin V staining and flow cytometry. At 72 h after transfection, the number of annexin V-positive apoptotic cells was decreased by ANP32B knockdown in both Huh7 and HLE cells treated with staurosporine (Fig 1C). These data indicate that ANP32B downregulation suppresses apoptosis by affecting the mitochondrial pathway.

### Silencing of ANP32B altered expression of Bak and phosphorylation of Bad, two apoptosis-related genes of the BCL-2 family

**Fig 2:**
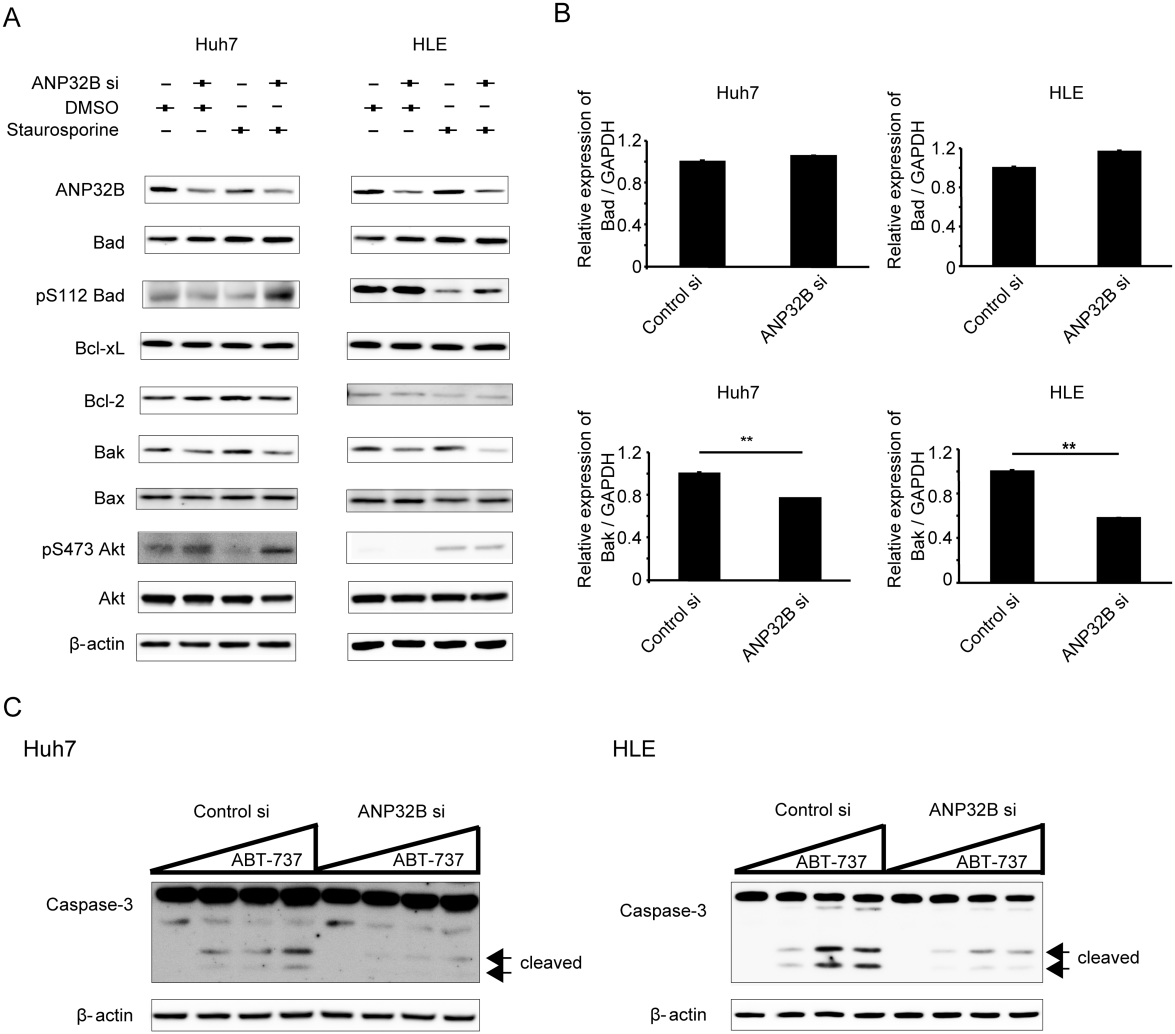
Silencing of ANP32B regulated Bak expression and the phosphorylation of Bad. **(A)** Expression of genes involved in the mitochondrial pathway and Akt; phosphorylated Akt was analyzed by Western blotting after ANP32B knockdown (left panels; Huh7 cells, right panels; HLE cells). **(B)** The altered levels of BAD and BAK mRNA expression were analyzed by real time RT-PCR using ANP32B siRNA (left panels; Huh7 cells, right panels; HLE cells). Mean ± SEM of six replicates. **p < 0.01. **(C)** Huh7 and HLE cells presenting ANP32B downregulation were cultured for 4 h with increasing concentrations (0, 1, 4, and 8 μM) of ABT-737, an anti-apoptotic agent. The expression of cleaved caspase-3 was analyzed by Western blotting.

Bcl-2 family members are regulators of apoptosis in the mitochondrial pathway [19,20]. Among the Bcl-2 family members, the Bcl-2 homology (BH) 3-only proteins are known to induce apoptosis by increasing mitochondrial outer membrane permeability [21]. To analyze the function of ANP32B in apoptosis, we first compared the expression profiles of genes involved in apoptosis by using real-time PCR arrays (S1 Table) and Pathscan (S1C Fig). We identified two apoptosis-related genes of the Bcl-2 family that were modulated by ANP32B (Fig 2A). Phosphorylated Bad was upregulated by ANP32B knockdown in cells treated with staurosporine when compared with cells expressing the control siRNA. In contrast, Bak was downregulated by ANP32B knockdown with or without staurosporine treatment (Fig 2A). It has been reported that ANP32B expression, which is positively correlated with phosphorylated Akt levels, regulates AKT activation in breast cancer [22]. Akt expression was not altered in both types of HCC cells; however, phosphorylated Akt was upregulated by ANP32B knockdown in Huh7 cells treated with staurosporine (Fig 2A). Real-time RT-PCR indicated that BAK mRNA was downregulated by ANP32B silencing. However, BAD mRNA expression was not altered (Fig 2B). We cultured Huh7 cells with etoposide, a pro-apoptotic agent, and knocked down ANP32B expression in Huh7 cells using another siRNA (si2ANP32B) (S2 Fig). Similar results were obtained. Moreover, Z-VAD-FMK was applied to cells before the apoptosis-inducing agent (staurosporine). The anti-apoptotic effect observed with knockdown of ANP32B was not observed (S3 Fig).

We then used ABT-737, which binds to and inhibits the anti-apoptotic proteins of the Bcl-2 family. The expression of cleaved forms of caspase 3 increased in a concentration-dependent manner after addition of ABT-737. However, this effect was attenuated by ANP32B silencing in both Huh7 and HLE cells. ANP32B silencing rendered HCC cells resistant to apoptosis even in culture with ABT-737 (Fig 2C).

### Overexpression of ANP32B altered the expression of apoptosis-related genes, but did not induce apoptosis

**Fig 3:**
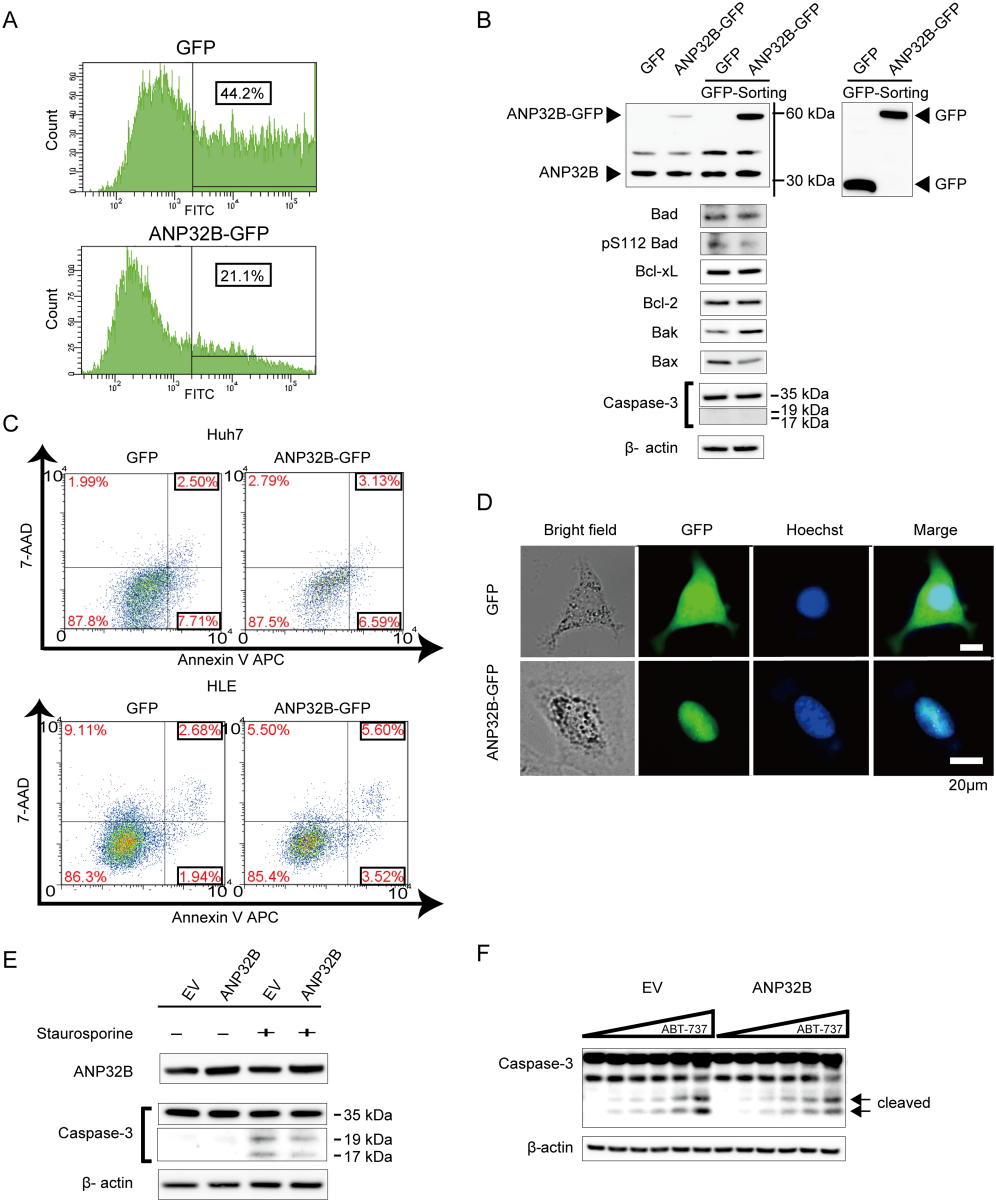
ANP32B overexpression using plasmids and lentiviruses expressing ANP32B. **(A)** Huh7 cells were transfected with GFP- and ANP32B-GFP-encoding plasmids. The number of GFP-positive cells represented the transfection efficiency. GFP-positive cells were collected by FACS sorting. **(B)** The collected GFP-positive cells expressed ANP32B at high levels as analyzed by Western blotting. The expression of some apoptosis-related genes of the Bcl-2 family was altered by ANP32B overexpression. However, the expression of cleaved caspase-3 was not altered. **(C)** Apoptotic effects of ANP32B overexpression were determined in collected GFP-positive cells using flow cytometry. Apoptotic cells were positively labeled with annexin V, and necrotic cells were labeled with 7-AAD (upper panels; Huh7 cells, lower panels; HLE cells). **(D)** Lentivirus expressing ANP32B was also used. Huh7 cells were infected with the lentivirus expressing ANP32B. The transduced cells expressing GFP were examined using fluorescence microscopy. The nucleus was stained with Hoechst33342. The scale bar indicates a length of 20 μm. **(E)** Huh7 cells infected with lentivirus expressing ANP32B were cultured with staurosporine. The expression of cleaved forms of caspase 3 protein was analyzed by Western blotting at 12 h after the addition of staurosporine. **(F)** Huh7 cells infected with lentivirus expressing ANP32B were cultured for 7 h with increasing concentrations (0, 0.5, 1, 2, 4, and 8 μM) of ABT-737, and the expression of cleaved caspase-3 was analyzed by Western blotting.

To upregulate ANP32B, we used pEZ-M03-ANP32B-GFP, which encodes the ANP32B gene (ANP32B-GFP) and used pEZ-M03-GFP as a control plasmid (GFP). Because the percentage of GFP-positive cells, which indicated successful transfection, varied in ANP32B-GFP- and GFP-transfected cells (21.1% vs. 44.2%, respectively) (Fig 3A), we collected only GFP-positive cells by FACS sorting to examine the effect of ANP32B overexpression. We then analyzed the apoptosis-related genes of the Bcl-2 family using the GFP-positive cells. In contrast to the data for ANP32B silencing, ANP32B overexpression decreased Bad phosphorylation and upregulated Bak. However, Bax was downregulated, and, as a result, ANP32B overexpression failed to induce an increase in cleaved caspase-3 levels (Fig 3B). Moreover, FACS analysis indicated that the number of annexin V-positive apoptotic cells did not increase after ANP32B overexpression in either Huh7 or HLE cells (Fig 3C).

Additionally, we used the lentiviral system to efficiently overexpress ANP32B. First, we used lentiviruses expressing cDNA encoding ANP32B-GFP or GFP and compared GFP expression within the cells by using fluorescence microscopy. The expression of GFP-labeled ANP32B was localized in the nucleus of hepatocytes. In contrast, the expression of control GFP was not localized, but distributed in the nucleus and cytoplasm (Fig 3D). To determine the effect of apoptosis, we compared Huh 7 cells infected with empty vector (EV) and those infected with ANP32B lentiviral vectors. ANP32B overexpression by lentiviral vectors also failed to induce apoptosis. The levels of the cleaved forms of caspase 3 were not altered by ANP32B overexpression using lentiviral vectors even in cells cultured with staurosporine or ABT-737 (Fig 3E and 3F). From these results, ANP32B overexpression could not induce apoptosis with or without the pro-apoptotic agent.

### ANP32B is expressed in human HCC tissue and low levels of ANP32B are correlated with advanced pathological stage

**Fig 4:**
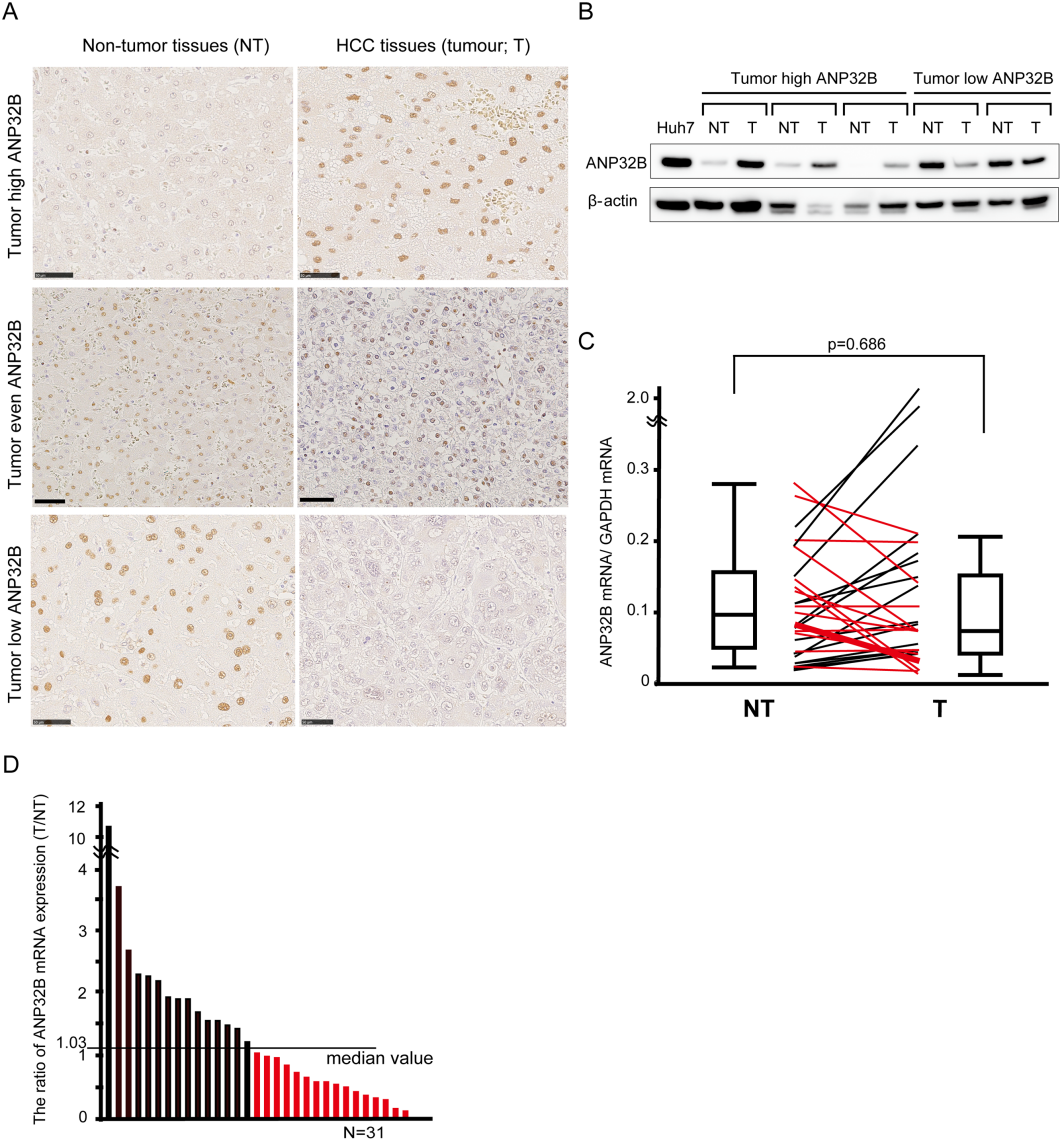
Expression of ANP32B in human HCC tissue. **(A)** Immunohistochemical staining of ANP32B in human HCC tissues (T) and non-tumor tissues (NT); the scale bar indicates a length of 50 μm. **(B)** Western blotting analysis of 3 patients with higher expression of ANP32B in T than in NT (tumor high ANP32B) and 2 patients with lower expression of ANP32B in T than in NT (tumor low ANP32B). **(C)** Levels of ANP32B mRNA in T and NT were compared individually. **(D)** Patients were divided into two groups by the T/NT ratio of ANP32B mRNA expression. Red bars represent the tumor low ANP32B group and black bars represent the tumor high ANP32B group. The cutoff value that divided the groups was 1.03.

To investigate the role of ANP32B expression in patients with HCC, immunohistochemical staining was performed to determine whether ANP32B is expressed in HCC tissues (T) and non-tumor tissues (NT) from surgical specimens. Immunohistochemical staining of NT and T tissues for ANP32B revealed that positive ANP32B signals localized to the nucleus of hepatocytes (Fig 4A). Upper paired photos titled “Tumor high ANP32B” show images of specimens from a patient who presented higher expression of ANP32B in HCC tissue than in non-tumor tissue. Middle paired photos show images of specimens from a patient who presented similar expression of ANP32B in HCC and non-tumor tissue. Lower paired photos show images of specimens from a patient who presented lower expression of ANP32B in HCC tissue than in non-tumor tissue. Western blot analysis also shows the ANP32B expression in T and NT tissues from 5 surgical specimens (Fig 4B).

We next calculated the copy number ratio of ANP32B mRNA/GAPDH mRNA, and compared this ratio between T and NT tissues (Fig 4C). No difference was observed between groups. Thus, we determined the mRNA expression index of T and NT specimens from each patient, calculated the T/NT ratio, and divided the patients into two groups: one with higher ANP32B expression (tumor high ANP32B) and another with lower ANP32B expression (tumor low ANP32B) in T than in NT (Fig 4D). The cutoff value to separate the groups was 1.03 (median value of the T/NT ratio). We list the clinicopathological features of the 31 enrolled patients with HCC in Table 1. Among the 31 patients, 16 belonged to the tumor low ANP32B group. The patients presenting tumor low ANP32B exhibited significantly advanced pathological stage relative to patients presenting tumor high ANP32B (S2 and S3 Tables).

**Table 1:** Clinicopathological parameters of patients with hepatocellular carcinoma (HCC) (N = 31).

	Tumor low ANP32B (N = 16)	Tumor high ANP32B (N = 15)	p-value
Age (mean ± SD), year	69 (49–81)	67 (51–83)	0.729
Sex (M/F)	12/ 4	12/ 3	0.739
Virus (HBV/HCV/none)	4/ 8/ 4	5/ 7/ 3	0.865
Child-Pugh (A/B/C)	16/ 0/ 0	14/ 0/ 1	0.223
Fibrosis (F1/ F2/ F3/ F4)	2/ 2/ 3/ 9	2/ 6/ 2/ 5	0.331
Pathological stage (I+II/ III+IV)	10/ 6	14/ 1	0.032*
AFP, ng/ml (range)	26.5 (2–1525)	7.2(1.6–936)	0.710
DCP, mAU/ml (range)	409 (16–418820)	65 (13–6012)	0.284
PT, % (range)	84.1 (72.8–100.8)	86.6 (56.5–180.9)	0.976
Tumor size	36 (15–160)	30 (18–160)	0.976
Tumor differentiation (well/ moderate/ poor)	2/ 12/ 2	4/ 10/ 1	0.557
Tumor multiplicity (solitary/ multiple)	12/ 4	9/ 6	0.371

* These *P*-values are significant.

HBV, hepatitis B virus; HCV, hepatitis C virus; AFP, alpha-fetoprotein; DCP, des-carboxyprothrombin; PT, prothrombin time.

**Fig 5:**
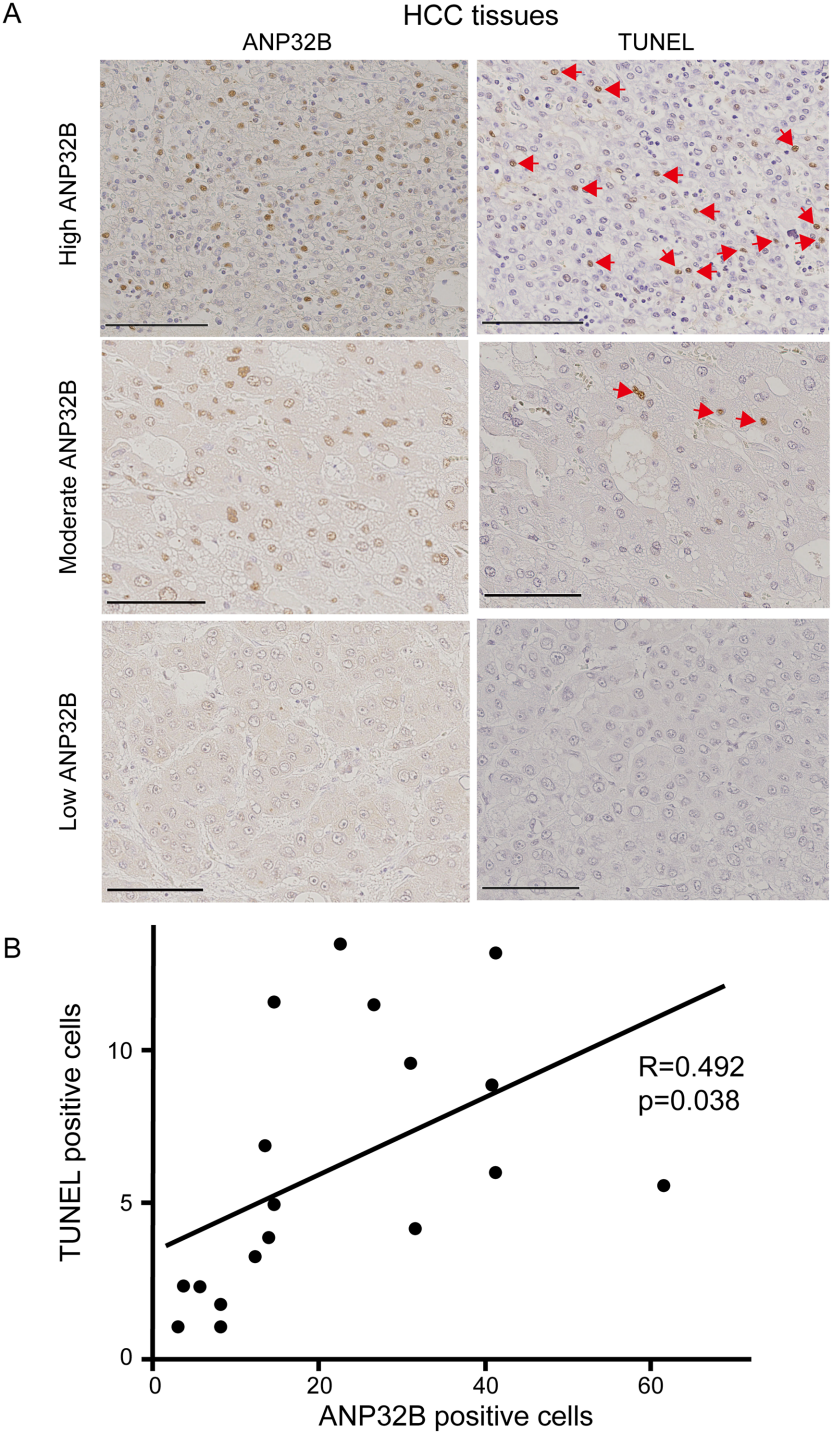
Relationship between ANP32B expression and apoptosis in human HCC tissues. **(A)** Immunohistochemical staining of ANP32B (left panels) and TUNEL staining of the liver sections (right panels) were analyzed. Patients were divided based on the expression levels of ANP32B, and the number of cells positive for TUNEL was compared. The scale bar indicates a length of 50 μm. Arrows indicate TUNEL-positive cells. **(B)** A significant correlation was observed between the expression of ANP32B and TUNEL positivity in HCC tissues (r = 0.492; p = 0.038).

Next, to investigate the significance of ANP32B in human HCC specimens, we evaluated the association between ANP32B expression and apoptotic cells, indicated by TUNEL positivity. TUNEL-positive cells were observed in parallel with ANP32B positivity (Fig 5A). There was a positive relationship between the expression of ANP32B and TUNEL positivity in 18 HCC tissues, as indicated in Fig 5B (r = 0.492; p = 0.038). These results indicate that downregulation of ANP32B could play an anti-apoptotic role in human HCC tissues.

## Discussion

This study is the first report confirming ANP32B expression in HCC and examining the role of ANP32B in this disease. Our study presents three major findings: first, the downregulation of ANP32B plays an anti-apoptotic role in HCC, as determined both in vitro and in vivo. Second, ANP32B inhibits Bax expression and the phosphorylation of Bad, while promoting Bak expression in the mitochondrial apoptosis pathway. Third, ANP32B is expressed in the nucleus of HCC cells, and low levels of ANP32B are related to advanced UICC stage of human HCC.

Apoptosis is an important pathway in the regulation of normal development and homeostasis in multicellular organisms [23]. In liver cancer, apoptosis is generally considered a tumor-preventing mechanism, as it removes deleterious cells with oncogenic alterations. Sensitivity to apoptosis influences tumor progression and metastasis, and determines how cancer cells respond to treatment [23,24]. However, several reports have shown that increased apoptosis of hepatocytes impairs liver homeostasis and induces hepatocyte proliferation and hepatocarcinogenesis [25,26]. In the present study, ANP32B downregulation suppressed apoptosis and was related to advanced UICC stage in patients with HCC; these findings were consistent with the previously reported mechanism.

In cancer, ANP32 has been reported to control cell death and regulate the activity of protein phosphatase and the epigenome [10]. To date, most reports have focused on ANP32A. The role of ANP32A in various cancers, including prostate [27], colorectal [28], and ovarian cancer [29], has been described. Furthermore, in HCC, ANP32A has been reported to promote growth and serve as a marker of poor prognosis related to apoptosis [4,5]. ANP32B has additionally been shown to have an anti-apoptotic function in leukemic cells [17,18]. However, Li et al. [16] reported that ANP32B inhibits the expression of the anti-apoptotic protein Bcl-2 and plays a pro-apoptotic role in leukemic cells. Recently, Yang et al. [22] reported that ANP32B deficiency decreases AKT phosphorylation, which is involves in regulating cell growth in breast cancer. Contrary to the findings of Yang et al., the present data indicate that phosphorylated Akt is upregulated following ANP32B knockdown in Huh7 cells treated with staurosporine. These data indicate that knockdown of ANP32B suppresses not only apoptosis but also cell proliferation. The present results suggest that ANP32B expression in breast cancer may differ from that in HCC.

**Fig 6:**
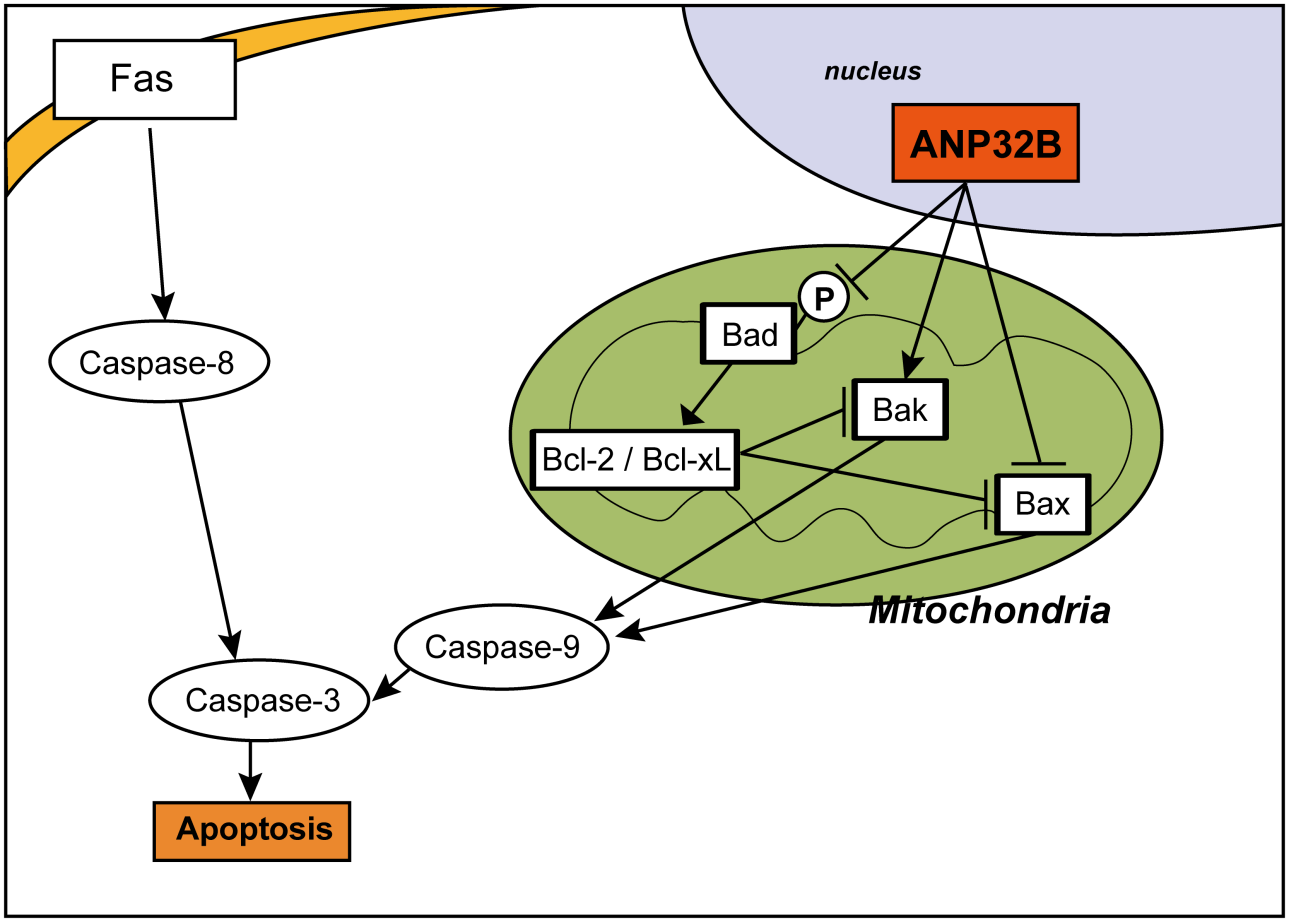
Proposed model illustrating the role of ANP32B and its relationship with other molecules in apoptosis. ANP32B inhibits Bax and phosphorylation of Bad, and promotes Bak expression in the mitochondrial apoptosis pathway.

We summarized our in vitro results to outline a model depicting the regulation of apoptosis by ANP32B via the modulation of Bak, Bax, and phosphorylated Bad in HCC (Fig 6). Downregulation of ANP32B increases the phosphorylation of Bad and decreased Bak expression, playing an anti-apoptotic role. In this situation, the expression of Bax does not increase because of the inhibition by Bcl-2 and Bcl-xL associated with the increase in Bad phosphorylation. In contrast, ANP32B overexpression decreases Bad phosphorylation and upregulated Bak, but does not induce apoptosis; Bax suppression occurs simultaneously. Thus, we hypothesize that high ANP32B expression is needed to maintain cellular homeostasis and prevent apoptosis.

Bak and Bax are members of the Bcl-2 family that promote the release of apoptotic factors from the mitochondria into the cytosol [30,31]. Among the BH3-only family of proteins, Bad is characterized by pro-apoptotic effects achieved through the neutralization of anti-apoptotic proteins such as Bcl-xL [21]. Bad function is under stringent negative regulation by phosphorylation [32]. Five phosphorylation sites (Ser112, Ser128, Ser136, Ser155, and Ser170) have been identified on Bad; these sites are phosphorylated by a variety of kinases [33]. Phosphorylation at Ser112 and Ser136 promotes the binding of Bad to 14-3-3 proteins to prevent association between Bad, Bcl-2, and Bcl-xL [33,34]. Therefore, phosphorylated Bad plays an anti-apoptotic role through Bcl-xL and Bcl-2. In our study, downregulation of ANP32B increased Bad phosphorylation at Ser112. Conversely, ANP32B overexpression decreased Bad phosphorylation at Ser112. These findings indicate that ANP32B acts as a kinase inhibitor for Bad phosphorylation.

This study demonstrates that a low tumor/non-tumor ratio of ANP32B mRNA expression is related to advanced UICC stage. In human HCC tissues, TUNEL-positive cells were observed in parallel with ANP32B expression. Downregulation of ANP32B in HCC may result in decreased sensitivity to apoptosis, thereby contributing to tumor progression. These findings suggest that ANP32B acts as a prognostic marker in HCC as a result of its relationship with apoptosis. Furthermore, ANP32B may serve as a therapeutic target for gene therapy of HCC. Suppression of the downregulation of ANP32B should therefore prevent the development of HCC. Moreover, identification of the factors that downregulate ANP32B may lead to the identification of targets to regulate the apoptosis of HCC related to ANP32B.

ABT-737 is a recently developed compound that binds to and inhibits the anti-apoptotic proteins of the Bcl-2 family, including Bcl-xL and Bcl-2, with a specificity similar to that of Bad. ABT-737 is one of the first reported small-molecule inhibitors of the Bcl-2 family proteins that target the apoptosis machinery, thus showing potential for the treatment of cancers including HCC [35–38]. In this study, we showed that silencing of ANP32B rendered HCC cells resistant to apoptosis, even when the cells were cultured with ABT-737. These data suggest that ANP32B silencing has anti-apoptotic effects by modulating phosphorylated Bad and the expression of Bak directly, and that its effects are not dependent on Bcl-2 and Bcl-xL. ABT-737 is expected to serve as a therapeutic drug for patients with HCC, and ANP32B could be useful as a biomarker for ABT-737 treatment. Sorafenib is currently the only effective pharmaceutical drug for HCC [39]. Galmiche et al. [37] reported that sorafenib activates Bad in HCC cells by regulating its expression and preventing its inactivation by phosphorylation. Downregulation of ANP32B increased the phosphorylation of Bad, playing an anti-apoptotic role. This finding indicates that ANP32B may also serve as a biomarker for sorafenib treatment. However, further investigation is needed to evaluate the clinical utility of ANP32B in the selection of these treatments.

This study suffers from some limitations. First, the molecules that downregulate ANP32B have not been identified. Second, the sample size for the clinical data was small. Finally, the results of the study on human tissues are expressed as the ratio of ANP32B expression between tumor and non-tumor tissues. This ratio may be affected by background factors. To overcome these limitations, further long-term prospective studies in a larger cohort are needed.

In conclusion, the current results demonstrate that ANP32B modulates Bad phosphorylation as well as Bak and Bax expression, thereby regulating apoptosis in HCC. In particular, ANP32B downregulation plays a role in the suppression of apoptosis. ANP32B may potentially serve as a therapeutic target for the treatment of HCC.

## Supporting information

S1 Fig ANP32B protein expression and ANP32B knockdown by siRNA in HCC cell lines. **(A)** ANP32B protein expression in HCC cell lines analyzed by Western blotting. β-actin was used as an internal control. Jurkat cells were used as a positive control. **(B)** ANP32B knockdown by siRNA in Huh7 cells at days 1 to 3 after transfection. Mean ± SEM of six replicates. **p < 0.01. **(C)** Huh7 and HLE cells were transfected with either ANP32B siRNA or control siRNA and analyzed by using the PathScan intracellular signaling array kit.
(TIF)

S2 Fig ANP32B knockdown by siRNA suppressed apoptosis in HCC cell lines. **(A)** ANP32B was knocked down by siRNA, and cells were cultured with the pro-apoptotic agent etoposide (600 μM for Huh7 cells) for 24 h. The expression of cleaved forms of caspase 3, caspase 8, caspase 9, and phosphorylated Bad and Bak proteins was analyzed by Western blotting. **(B)** ANP32B was knocked down by siRNA (ANP32B si2), and cells were cultured with staurosporine for 12 h. The expression of cleaved forms of caspase 3, caspase 8, and caspase 9 proteins was analyzed by Western blotting.
(TIF)

S3 Fig Effect of caspase inhibitor and ANP32B knockdown in HCC cell lines. Huh7 cells were transfected with ANP32B siRNA or control siRNA, and 30 μM Z-VAD-FMK was applied to cells before application of an apoptosis inducing agent (1 μM staurosporine). After 18 h of incubation, we analyzed apoptosis by annexin V staining with flow cytometry.
(TIF)

S1 Table PCR array analysis of the effect of downregulation of ANP32B on apoptosis-related genes. (DOCX)

S2 Table Laboratory data of patients with HCC. (DOCX)

S3 Table Tumor status in HCC. (DOCX)
