# Supplementary figures and images for: Downregulation of ANP32B exerts anti-apoptotic effects in hepatocellular carcinoma
Source: PLoS One. 2017 May 9;12(5):e0177343. doi: 10.1371/journal.pone.0177343 (PMC5423643; doi:10.1371/journal.pone.0177343)

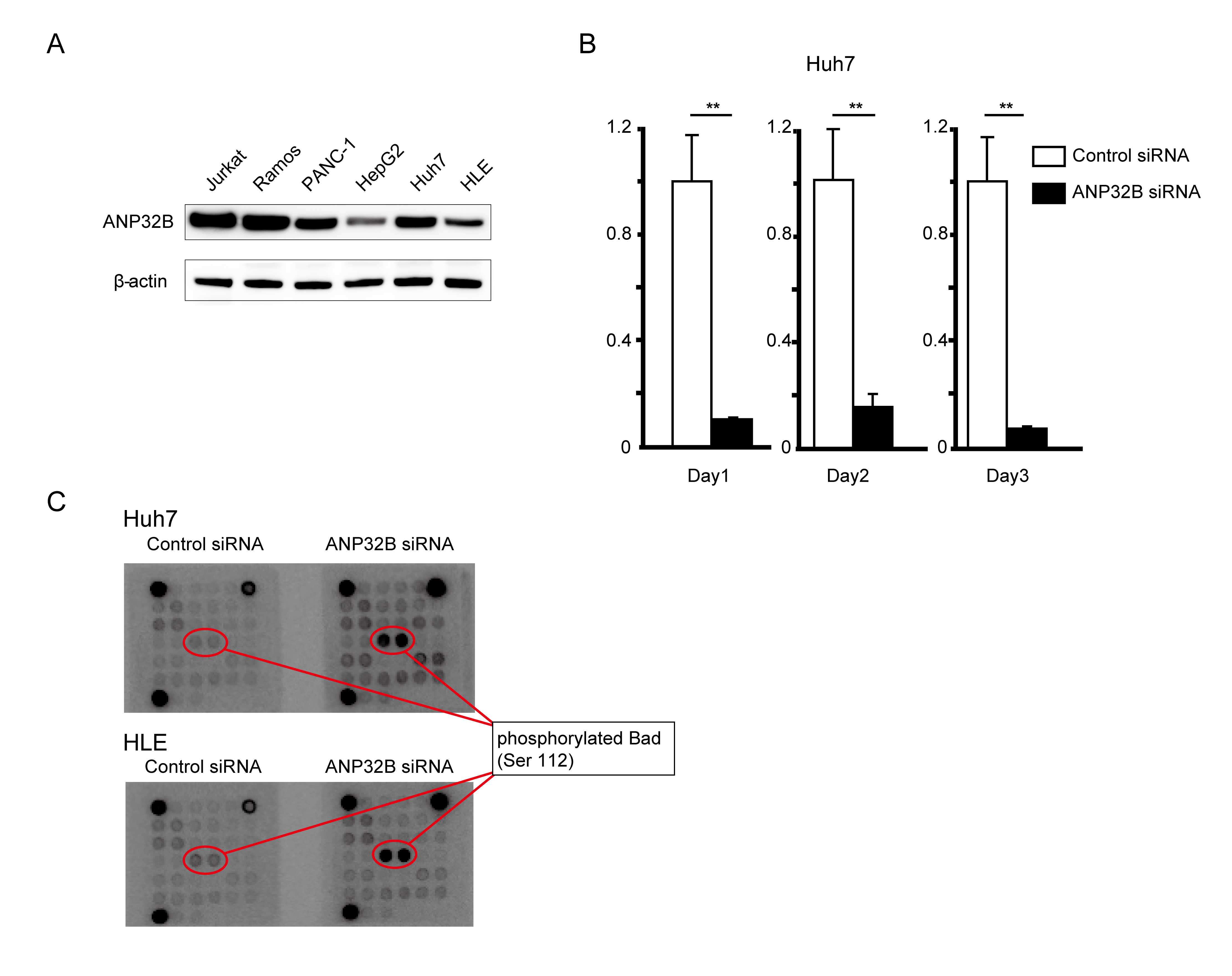

Supplement: S1 Fig — (A) ANP32B protein expression in HCC cell lines analyzed by Western blotting. β-actin was used as an internal control. Jurkat cells were used as a positive control. (B) ANP32B knockdown by siRNA in Huh7 cells at days 1 to 3 after transfection. Mean ± SEM of six replicates. **p < 0.01. (C) Huh7 and HLE cells were transfected with either ANP32B siRNA or control siRNA and analyzed by using the PathScan intracellular signaling array kit. (TIF) [file pone.0177343.s001.tif]

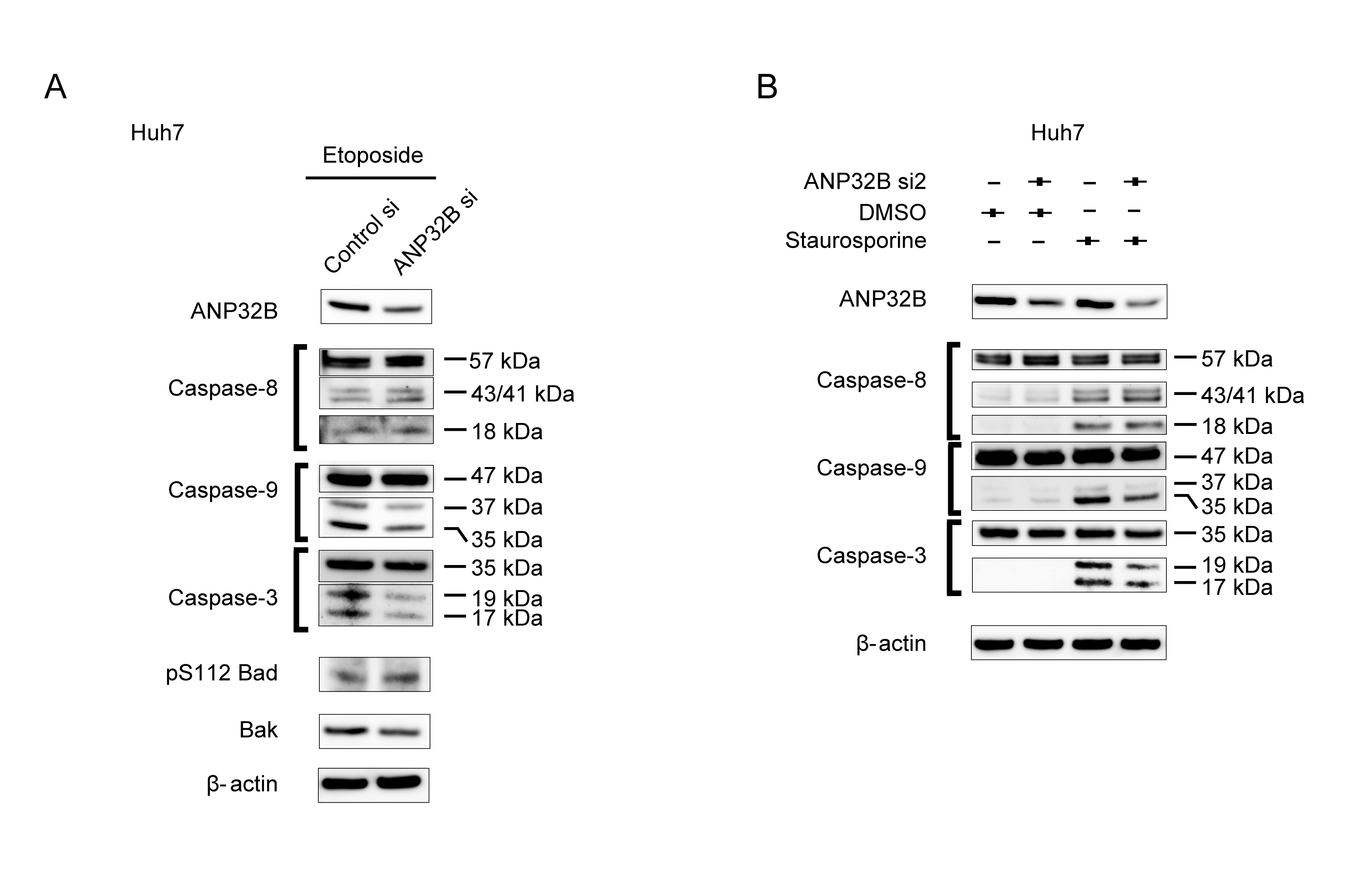

Supplement: S2 Fig — (A) ANP32B was knocked down by siRNA, and cells were cultured with the pro-apoptotic agent etoposide (600 μM for Huh7 cells) for 24 h. The expression of cleaved forms of caspase 3, caspase 8, caspase 9, and phosphorylated Bad and Bak proteins was analyzed by Western blotting. (B) ANP32B was knocked down by siRNA (ANP32B si2), and cells were cultured with staurosporine for 12 h. The expression of cleaved forms of caspase 3, caspase 8, and caspase 9 proteins was analyzed by Western blotting. (TIF) [file pone.0177343.s002.tif]

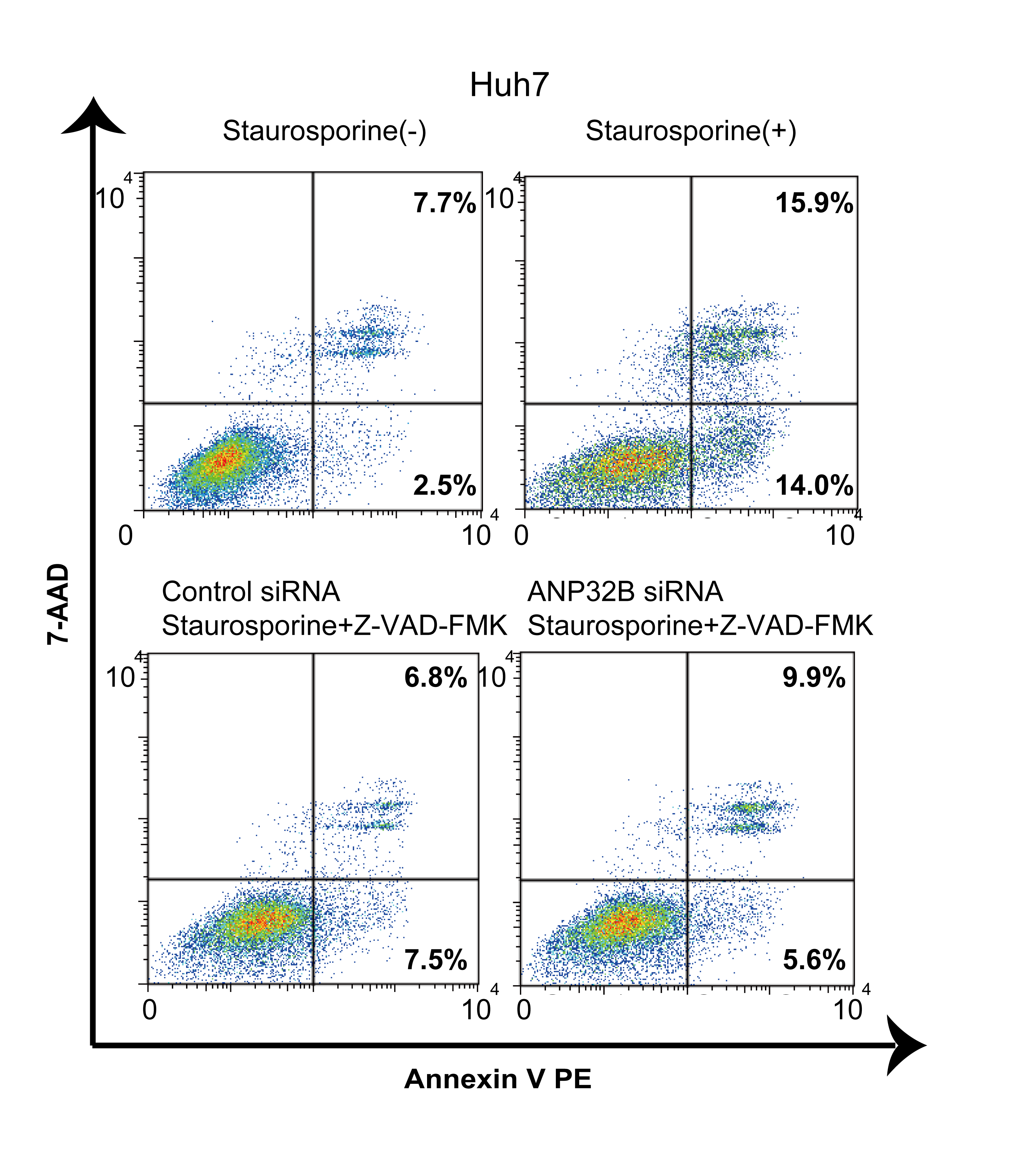

Supplement: S3 Fig — Huh7 cells were transfected with ANP32B siRNA or control siRNA, and 30 μM Z-VAD-FMK was applied to cells before application of an apoptosis inducing agent (1 μM staurosporine). After 18 h of incubation, we analyzed apoptosis by annexin V staining with flow cytometry. (TIF) [file pone.0177343.s003.tif]
