# Supplementary material for: Downregulation of ANP32B exerts anti-apoptotic effects in hepatocellular carcinoma
Source: PLoS One. 2017 May 9;12(5):e0177343. doi: 10.1371/journal.pone.0177343 (PMC5423643; doi:10.1371/journal.pone.0177343)
Supplement: S1 Table — (DOCX) [file pone.0177343.s004.docx]

**Table S1. PCR array analysis of the effect of down-regulated ANP32B on apoptosis-related genes**

| Symbol | Fold Up- or Down-Regulation |  | Symbol | Fold Up- or Down-Regulation |
| --- | --- | --- | --- | --- |
| AIFM1 | 1.18 |  | CASP8 | 1.10 |
| AKT1 | -1.36 |  | DFFA | -1.11 |
| BAD | -1.23 |  | HRK | -2.10 |
| BAK1 | -1.45 |  | LTBR | -1.27 |
| BCL10 | -1.17 |  | NAIP | -1.22 |
| BCL2L1 | -2.00 |  | NOD1 | -1.21 |
| BCL2L11 | -1.78 |  | RIPK2 | -1.17 |
| BIK | -1.21 |  | TP53 | -1.15 |
| BIRC3 | 1.69 |  | TP53BP2 | -1.24 |
| BNIP3L | 1.66 |  | TRAF3 | -1.28 |
| BRAF | -1.19 |  | ACTB | -1.12 |
| CASP1 | -1.35 |  | B2M | -1.18 |
| CASP10 | -1.19 |  |  | |
| CASP3 | -1.38 |  |  |  |
| CASP6 | -1.19 |  |  |  |
