# Supplementary material for: Downregulation of ANP32B exerts anti-apoptotic effects in hepatocellular carcinoma
Source: PLoS One. 2017 May 9;12(5):e0177343. doi: 10.1371/journal.pone.0177343 (PMC5423643; doi:10.1371/journal.pone.0177343)
Supplement: S2 Table — (DOCX) [file pone.0177343.s005.docx]

**Table S2. Laboratory data of patients with HCC**

|  | **Tumor low ANP32B (N = 16)** | **Tumor high ANP32B (N = 15)** | **p-value** |
| --- | --- | --- | --- |
| Platelets (10^4^/μl) | 15.8 (6.3-27.2) | 14.2 (6.7-38.9) | 0.524 |
| Albumin (g/dl) | 3.8 (3.2-4.5) | 3.7 (2.9-4.5) | 0.785 |
| γ-Glutamyl transpeptidase (U/l) | 48 (24-321) | 41 (18-812) | 0.606 |
| Alkaline phosphatase (U/l) | 256 (170-607) | 269 (181-619) | 0.548 |
| Ferritin (ng/ml) | 128 (9.1-243) | 137 (11-923) | 0.350 |
| Total bilirubin (mg/dl) | 0.7 (0.4-1.3) | 0.7 (0.4-2) | 0.870 |
| Aspartate aminotransferase (U/l) | 40 (24-118) | 38 (20-106) | 0.248 |
| Alanine aminotransferase (U/l) | 44 (16-164) | 31.5 (15-228) | 0.997 |
| Cholinesterase (U/l) | 133 (88-315) | 175 (66-336) | 0.610 |
| Total cholesterol (mg/dl) | 156.5 (113-236) | 173 (111-258) | 0.323 |
| Type Ⅳ collagen 7S (ng/ml) | 6.5 (2-15) | 6.0 (3.8-12) | 0.617 |
| Hyaluronic acid (ng/ml) | 171.5 (32-621) | 109.5 (17-1740) | 0.635 |
| AFP-L3 positive | 3/13 | 5/14 | 0.470 |
