# Supplementary material for: Downregulation of ANP32B exerts anti-apoptotic effects in hepatocellular carcinoma
Source: PLoS One. 2017 May 9;12(5):e0177343. doi: 10.1371/journal.pone.0177343 (PMC5423643; doi:10.1371/journal.pone.0177343)
Supplement: S3 Table — (DOCX) [file pone.0177343.s006.docx]

**Table S3. Tumor status in HCC**

|  | **Tumor low ANP32B (N = 16)** | **Tumor high ANP32B (N = 15)** | **p-value** |
| --- | --- | --- | --- |
| Expansive / Infiltrative growth (Eg/Ig) | 10/2 | 11/2 | 0.9304 |
| Formation of capsule (Fc) | 10/13 | 11/13 | 0.618 |
| Infiltration to capsule (Fc-Inf) | 6/13 | 8/10 | 0.092 |
| Septal formation (Sf) | 9/13 | 9/12 | 0.748 |
| Intrahepatic metastasis (IM) | 2/11 | 0/10 | 0.096 |
| Vascular invasion (Vp, Vv, B) | 5/13 | 4/12 | 0.789 |
